# Perceptions of Orthopedic and Musculoskeletal Practice Among Primary Care Physicians in Japan: A Questionnaire Survey Analysis of Jichi Medical University Graduates

**DOI:** 10.7759/cureus.76236

**Published:** 2024-12-23

**Authors:** Ryo Sugawara, Ryusuke Ae, Shuhei Hiyama, Yasuhiro Higai, Naoki Yamaguchi, Hiroko Tomizawa, Katsushi Takeshita

**Affiliations:** 1 Department of Orthopaedics, Jichi Medical University, Shimotsuke, JPN; 2 Division of Public Health, Center for Community Medicine, Jichi Medical University, Shimotsuke, JPN

**Keywords:** medical education system, musculoskeletal diseases, orthopedics, primary medical care, training program

## Abstract

Background

Primary care physicians (PCPs) are expected to engage in comprehensive medical care, including orthopedic and musculoskeletal problems. This study aimed to assess perceptions and current status regarding orthopedic and musculoskeletal practice among PCPs in Japan.

Methodology

A cross-sectional survey was conducted among PCPs who graduated from Jichi Medical University (JMU) with opportunities to treat orthopedic and musculoskeletal problems. A specific questionnaire was used to investigate perceptions of orthopedic and musculoskeletal practice. Participants were divided into groups based on their workplace (rural and remote areas/other areas) and length of career (<10 years and ≥10 years). The answers between these groups were statistically analyzed by using chi-square tests.

Results

Among 298 respondents, 121 (40.6%) were engaged in rural and remote areas and 69 (23.2%) had a career length of <10 years. Two hundred and eighty-one PCPs (94.3%) required orthopedic knowledge and procedural skills and mental burden was reported by 166 PCPs (55.7%). PCPs in rural and remote areas with careers <10 years in length required significantly more orthopedic knowledge and skills and were more willing to learn them. Also, they perceived a burden because of a lack of knowledge and procedural skills. Many PCPs could perform common procedures such as general care for trauma but were not confident in treating orthopedic-specific injuries such as fractures and dislocation.

Conclusions

PCPs in rural and remote areas with careers <10 years in length had the most varied problems in orthopedic and musculoskeletal practice. A new approach that can provide musculoskeletal and orthopedic knowledge and skills may be needed for PCPs.

## Introduction

Population aging has become an increasing global concern in recent years. To maintain a sustainable and effective healthcare system that is responsive to social needs in the context of population aging, there has been increasing momentum for primary care physicians (PCPs) to engage in comprehensive medical care because of the positive impact of this approach on various health outcomes [1-3]. Moreover, musculoskeletal disorders are a key cause of morbidity in elderly people. To prolong healthy life expectancy, PCPs are expected to be acquainted with orthopedic and musculoskeletal problems, especially in non-urban areas where a population is aging rapidly. Among countries with aging populations, Japan has experienced markedly rapid aging during the last several decades, with the world’s highest life expectancy alongside declining birthrates [4]. The Japanese Medical Specialty Board has recently established a new residency program for physicians specializing in general medicine (i.e., general practitioners [GPs]) to improve primary healthcare for the national population [2,5]. However, the shortage of PCPs and a growing urban-rural disparity in physician supply are still serious and enduring problems [2,6,7].

Although PCPs typically have expertise in general practice and general internal medicine, they have to see a large number of patients with orthopedic and musculoskeletal problems, which is even more pronounced in rural and remote settings [8-10]. Nevertheless, previous studies have shown that PCPs often possess insufficient orthopedic knowledge and lack confidence regarding the treatment of orthopedic and musculoskeletal problems, largely due to an inadequate medical school curriculum [11,12]. Recently, training in musculoskeletal health has been provided in primary care postgraduate training programs in many countries [13-16]. However, a sustainable and validated training program for non-orthopedic surgeons and PCPs has not been established in Japan because these training courses are held infrequently and for shorter periods. Therefore, we hypothesized that PCPs in Japan may not be sufficiently prepared to address orthopedic and musculoskeletal problems, which may be affecting their practice. Additionally, this may vary by years of experience and geographical regions. It is important to evaluate the perception and current status of orthopedic and musculoskeletal practice among PCPs in Japan.

To the best of our knowledge, few studies have investigated whether PCPs can adequately address primary orthopedic and musculoskeletal problems in their daily practice [11,12]. Furthermore, the work environment regarding orthopedic and musculoskeletal practice among PCPs has not been assessed. This study aimed to assess perceptions and current status regarding orthopedic and musculoskeletal practice among PCPs in Japan and, if there are problems, to consider how to improve them.

This article was previously presented as a meeting abstract at the 2022 Japanese Orthopedic Association Annual Meeting from May 19 to 22, 2022.

## Materials and methods

Design, setting, and participants

This study was planned in accordance with the regulations of the Jichi Medical University (JMU) Clinical Research Ethics Committee and approved (Approval ID: 21-048).

We conducted a cross-sectional study among Japanese PCPs who currently had opportunities to treat orthopedic, traumatic, or musculoskeletal problems in their daily practice. We focused on PCPs with experience in daily practice in rural and remote settings. Participants in this survey were identified as the graduates of JMU, representing PCPs with experience engaging in rural practice. The list of JMU graduates was provided by the JMU community medicine promotion section.

JMU is the only medical school in Japan whose mission is to produce rural physicians [17-19]. This university was established in 1972 with support from the government and all 47 prefectures in Japan to educate physicians of ethical standing to provide highly skilled medical care in rural and remote regions of Japan and to promote the health and well-being of people living in rural communities. All graduates of JMU are obliged to work mainly in rural regions for nine years of their career despite their postgraduation work preferences, and some post-obligation graduates also continue to practice in rural regions. 

This questionnaire survey was conducted from August 1 to 31, 2021. The inclusion criteria for this survey were graduates of JMU, non-orthopedic physicians (typically GPs and those engaged in general internal medicine), and physicians whose affiliated institutions were small hospitals (with 20-99 beds) or clinics without a regular physician specialized in orthopedics (orthopedic surgeons). The exclusion criteria were junior resident doctors with ≤2 years of career length and orthopedic surgeons. A request to participate in this survey and survey questionnaire was sent by mail to the target participants. Participants who agreed to take part in the survey returned their questionnaire answers by mail or online.

Variable definitions

Questionnaires measured demographic variables and included specific questions to investigate perceptions of orthopedic and musculoskeletal practice in PCPs. Demographic variables included career length as a physician, current workplace (rural and remote area/other areas), facility type (clinic/small hospital), and current specialty (GP/ internal medicine/others). Rural and remote areas are defined as mountainous areas, remote islands, and other areas where transportation conditions are unfavorable and it is difficult to secure medical care as following previous study [17], and the workplace in this survey meets this definition.

The specific questions are listed in Table 1. The specific questions were developed by authors who had experience engaging in rural practices because we were unable to identify previous questionnaire studies examining this issue. The primary topics of the specific questions comprised eight categories: orthopedic practice needs, needs for orthopedic knowledge and skills, physical burden, mental burden, unfavorable experiences caused by their orthopedic practice, interest in orthopedics, willingness to learn orthopedic knowledge and skills, and willingness to participate in orthopedic training sessions. These questions were answered using a five-point scale (1, very often/so many; 2, often/many; 3, sometimes/some; 4, rarely/few; 5, never/not at all) to perform an overall detailed evaluation. Also, responses of 1+2+3 were classified as *Yes* and responses of 4+5 were classified as *No* when making comparisons between target groups to simplify data analysis. For the question about the physical and mental burden, we assumed that physical burden meant the feeling of physical exhaustion caused by orthopedic treatment (e.g., a large number of patients, variety of cases, and practice). Also, mental burden was assumed to be the increase in mental stress caused by orthopedic treatment (e.g., a lack of knowledge or skills, and problems with interpersonal relationships). We included additional questions regarding the reasons for the burden as follows: lack of knowledge, lack of procedural skills, large number of patients, need for a longer time of practice, lack of therapeutic effect, need for urgent treatment, and other reasons. For the question about unfavorable experiences caused by their orthopedic practice, we also included additional questions regarding reasons as follows: exacerbation of symptoms, decrease in activities of daily living, trouble with patients/family, deterioration of life prognosis, trouble with other hospitals, and the details can be freely described.

**Table 1:** Questionnaire: Perceptions of orthopedic practice in primary care settings.

Specific questions
1. Orthopedic practice needs
Are there orthopedic practice needs in your daily practice?
2. Needs for orthopedic knowledge and skills
Are orthopedic knowledge and procedural skills required in your daily practice?
3. Physical burden
Do you feel a physical burden in orthopedic treatment?
4. Mental burden
Do you feel a mental burden in orthopedic treatment?
5. Unfavorable experience in orthopedic practice
Do you have unfavorable experiences caused by your own orthopedic practice?
6. Interest in orthopedic practice
Do you have an interest in orthopedic practice?
7. Willingness to learn orthopedic knowledge and skills
Do you want to learn orthopedic knowledge and procedural skills?
8. Willingness to participate in orthopedic training sessions
Do you want to participate in orthopedic training sessions or workshops?

In addition to these specific questions, the questionnaires contained questions about specific orthopedic and musculoskeletal practices (diseases/injuries and procedures/treatments), as listed in Table 2, to assess whether PCPs were capable of treating these conditions by themselves, and willing to learn more about relevant practices. While some of these procedures are required at the specialist level, many of the procedures were designed to be within the capabilities of non-specialists to know whether PCPs could provide proper first aid and refer to orthopedic specialists if needed.

**Table 2:** Questionnaire: Specific orthopedic diseases and injuries, procedures, and treatments (Answers: Can treat by myself/Willingness to learn more). ^†^Induced by metabolic disorders such as hyperuricemia and crystal arthritis. ^¶^General trauma such as incised wounds, lacerations, abrasions, and contusions. ^‡^Induced by general inflammatory disorders such as rheumatoid arthritis and spondylarthritis. ^§^Suture, debridement, and drainage. ^||^Reduction and external fixation for fracture or dislocation.

Specific orthopedic diseases and injuries	Orthopedic procedures and treatments
Metabolic diseases^†^	General care for trauma^§^
Osteoporosis	Joint puncture
Traumatic injury^¶^	Osteoporotic treatment
Osteoarthritis	Trigger block
Locomotive syndrome	Neurological examination
Fracture/dislocation	Orthopedic first aid^||^
Inflammatory disease^‡^	Joint examination
Peripheral nerve disease	Hydro release
Lumbar spine disease	Orthopedic image reading
Cervical spine disease	Sports injury treatment
Sports injury	Rehabilitation
Others	Orthopedic ultrasound
	Nerve block
	Caudal block

Statistical analysis

First, we described the distributions of answers for eight primary specific questions to assess perceptions of orthopedic practice in the daily care of PCPs. Second, we compared these answers among PCPs whose current workplaces were rural/other areas and with careers that were <10 and ≥10 years in length based on the hypothesis that perceptions of orthopedic practice in primary care settings differ by the nine-year obligation of JMU graduates and workplace. All missing variables were excluded from the analysis. Proportions of answers among these four groups were compared using chi-square tests. The accepted level of significance was a *P*-value < 0.05. Statistical analysis was performed using SPSS software (Version 26.0. IBM Corp., Armonk, NY). Finally, we investigated which specific orthopedic practices (diseases/injuries and procedures/treatments) PCPs reported being able to treat by themselves and willing to learn more about.

## Results

We picked up 783 PCPs who met the inclusion criteria from more than 4,000 JMU graduates, and 298 (38.1%) responded to the survey. Demographic characteristics of PCPs in this survey are shown in Table 3. Among the PCPs, 121 (40.6%) were engaged in rural and remote healthcare, and 69 (23.2%) had a career length of <10 years (within obligation service). Table 4 shows the answers to eight primary specific questions. Most PCPs reported that orthopedic practices were required in their daily practice (280 Yes, 94.0%). Orthopedic knowledge and procedural skills were also seen as required by most PCPs (281 Yes, 94.3%). The proportion of PCPs who experienced mental burden (166, 55.7%) was larger than the proportion who experienced physical burden (122, 40.9%). Also, approximately one-quarter of PCPs had experienced unfavorable experiences caused by their own orthopedic practice in primary care (76 Yes, 25.5%). Two hundred and sixty PCPs (87.2%) had an interest in orthopedic practice, and most of them were willing to learn orthopedic knowledge and skills and participate in orthopedic training sessions.

**Table 3:** Demographic characteristics (N = 298). ^†^With 20-99 beds (small hospitals). ^¶^Some physicians responded to have multiple specialties; therefore, the number totals do not much the total numbers and percentages do not total 100%.

Characteristic	*n* (%)
Career length of primary care	
<10 years	69 (23)
≥10 years	229 (77)
Current workplaces	
Rural areas	121 (41)
Other than rural areas	177 (59)
Facility types	
Clinics	223 (75)
Small hospitals^†^	75 (25)
Current specialties^¶^	
General practitioner	135 (45)
Internal medicine	158 (53)
Others	73 (24)

**Table 4:** Answers for eight primary specific questions and each number/percentage. Responses of 1+2+3 were classified as *Yes*, and responses of 4+5 were classified as *No* when making comparisons between target groups.

Specific questions	1. Very often/very many	2. Often/many	3. Sometimes/some	4. Rarely/few	5. Never/not at all	N/A
	Counted *Yes*			Counted *No*		
1. Orthopedic practice needs	141 (47.3%)	92 (30.9%)	47 (15.8%)	18 (6.0%)	0 (0%)	0 (0%)
	280 (94.0%)			18 (6.0%)		
2. Needs for orthopedic knowledge and skills	124 (41.6%)	100 (33.6%)	57 (19.1%)	17 (5.7%)	0 (0%)	0 (0%)
	281 (94.3%)			17 (5.7%)		
3. Physical burden	8 (2.7%)	46 (15.4%)	68 (22.8%)	117 (39.3%)	28 (9.4%)	31 (10.4%)
	122 (40.9%)			145 (48.7%)		
4. Mental burden	12 (4.0%)	74 (24.8%)	80 (26.8%)	92 (30.9%)	7 (2.3%)	33 (11.1%)
	166 (55.7%)			99 (33.2%)		
5. Unfavorable experience in orthopedic practice	1 (0.3%)	14 (4.7%)	61 (20.5%)	191 (64.1%)	29 (9.7%)	2 (0.7%)
	76 (25.5%)			220 (73.8%)		
6. Interest in orthopedic practice	66 (22.1%)	111 (37.2%)	83 (27.9%)	37 (12.5%)	1 (0.3%)	0 (0%)
	260 (87.2%)			38 (12.8%)		
7. Willingness to learn orthopedic knowledge and skills	83 (27.9%)	80 (26.8%)	80 (26.8%)	48 (16.1%)	4 (1.3%)	3 (1.0%)
	243 (81.5%)			52 (17.4%)		
8. Willingness to participate in orthopedic training sessions	107 (35.9%)	155 (52.0%)	0 (0%)	31 (10.4%)	4 (1.3%)	1 (0.3%)
	262 (87.9%)			35 (11.7%)		

The comparisons among PCPs divided according to the current workplaces and career length are shown in Table 5. Among these four groups, PCPs in rural and remote areas with careers <10 years in length required significantly more orthopedic knowledge and skills in their daily practice. Also, they were significantly more likely to be willing to learn orthopedic knowledge and skills and participate in orthopedic training sessions. Regarding PCPs with careers ≥10 years in length, those in rural and remote areas significantly required more orthopedic practice in their daily practice than those in other areas. PCPs in other areas with careers <10 years in length had significantly less interest in orthopedic practice than other groups. There were no significant differences among these groups in the percentage of physical/mental burden and unfavorable experiences. 

**Table 5:** Comparisons of perceptions for orthopedic practice in primary care physicians. Proportions of answers among these four groups were compared using chi-square tests. ^†^Numbers and percentage of *Yes* responses for each question. ^*^P < 0.05. ^**^P < 0.01.

Workplaces	Rural and remote areas		Other areas	
Career length of primary care	<10 years (n = 56)	≥10 years (n = 65)	<10 years (n = 13)	≥10 years (n = 164)
Questions	n (%)^†^			
1. Orthopedic practice needs	55 (98.2)	65 (100)**	13 (100)	147 (89.6)*
2. Needs for orthopedic knowledge and skills	56 (100)*	64 (98.5)	12 (92.3)	149 (90.9)*
3. Physical burden	28 (50)	29 (44.6)	5 (38.5)	60 (36.6)
4. Mental burden	36 (64.3)	39 (60)	7 (53.8)	84 (51.2)
5. Unfavorable experience caused by own orthopedic practice	19 (33.9)	16 (24.6)	1 (7.7)	40 (24.4)
6. Interest in orthopedic practice	48 (85.7)	61 (93.8)	9 (69.2)*	143 (87.2)
7. Willingness to learn orthopedic knowledge and skills	52 (94.5)**	58 (89.2)	12 (92.3)	121 (73.8)**
8. Willingness to participate in orthopedic training sessions	54 (96.4)*	60 (92.3)	12 (92.3)	137 (83.5)**

**Figure 1:**
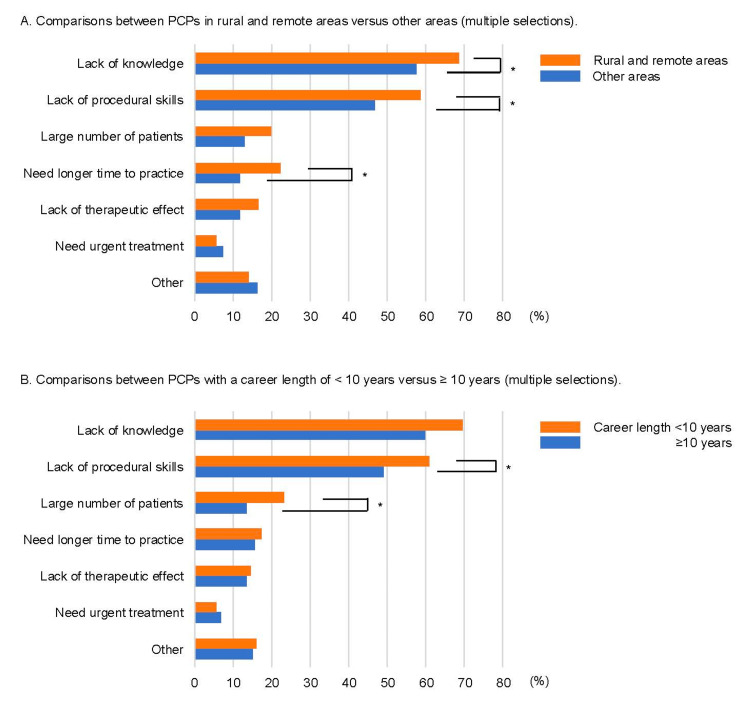
Reasons for the burden of orthopedic practice in primary care settings. The percentage for each item represents a percentage of agreement of those who answered *Yes* to the question: Do you feel a burden in orthopedic treatment? (A) Comparisons between PCPs in rural and remote areas vs. other areas (multiple selections). (B) Comparisons between PCPs with <10 vs. ≥10 years of career length (multiple selections). ^*^P < 0.05.

**Figure 2:**
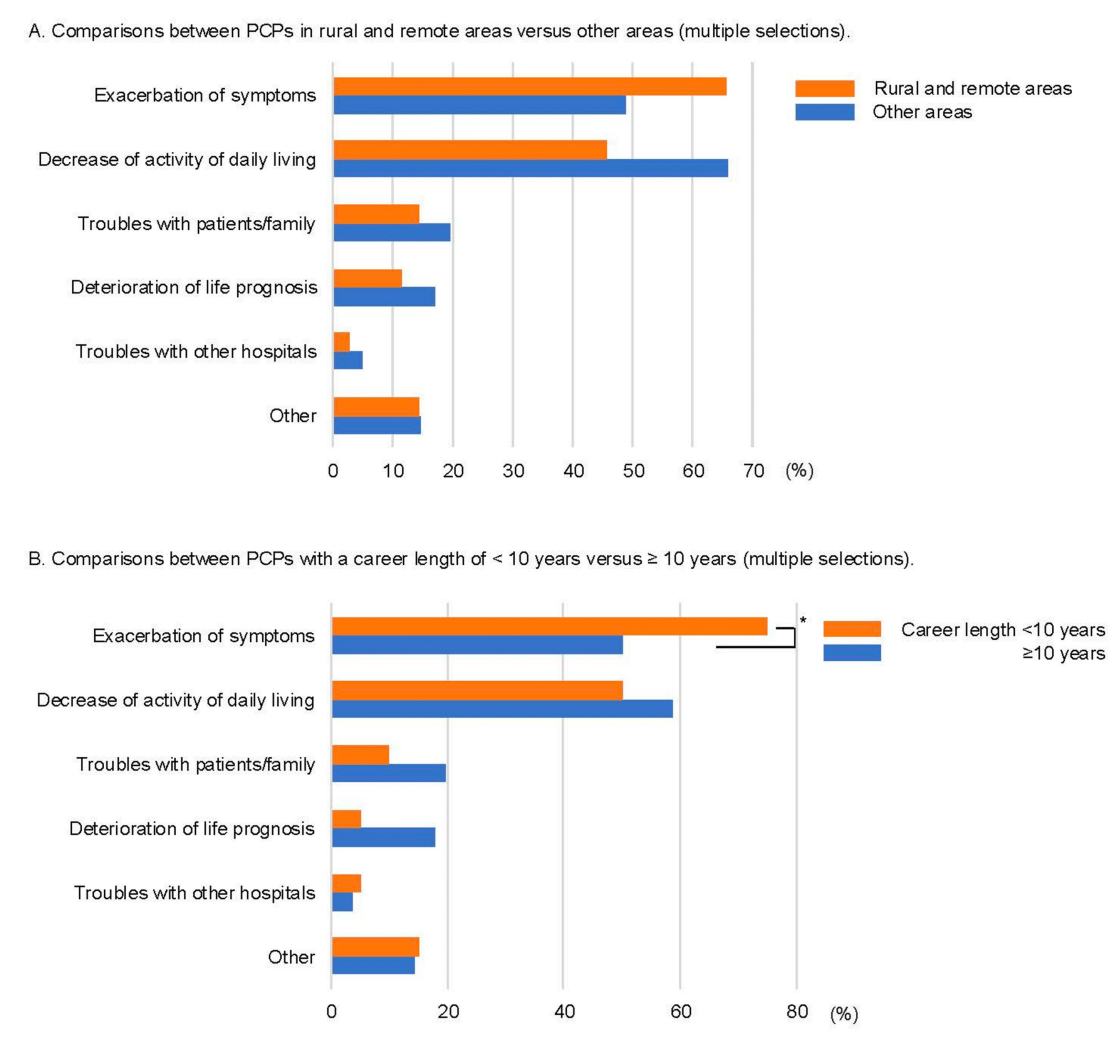
Reasons for unfavorable experience in orthopedic practice in primary care settings. The percentage for each item represents a percentage of agreement of those who answered *Yes* to the question: Do you have unfavorable experiences caused by your own orthopedic practice? (A) Comparisons between PCPs in rural and remote areas vs. other areas (multiple selections). (B) Comparisons between PCPs with <10 vs. ≥10 years of career length (multiple selections). ^*^P < 0.05.

The reasons for burdens in orthopedic practice are shown in Figure 1. Although common reasons included a lack of orthopedic knowledge and procedural skills, PCPs in rural and remote areas and those with careers that were <10 years in length were significantly more likely to perceive burden because of a lack of knowledge and procedural skills. On the other hand, not many PCPs indicated other reasons (e.g., a large number of patients, need longer time to practice) as the cause of their burden. Additionally, the majority of unfavorable experiences caused by their own orthopedic practice were exacerbations of symptoms (Figure 2). Compared with PCPs with careers ≥10 years in length, those with careers <10 years in length had significantly more experiences of exacerbation of symptoms (*n* = 114, 50.0% vs. *n* = 51, 75.0%, respectively). Also, PCPs in rural and remote areas had more such experiences than those in other areas without significant differences. From the free description, the main reasons for the exacerbation of symptoms were potential misdiagnosis and inappropriate treatments. For example, some PCPs experienced missed fractures, congenital diseases, and bone tumors. Others were transported to the mainland because they were unable to perform a reduction for fracture or dislocation.

**Figure 3:**
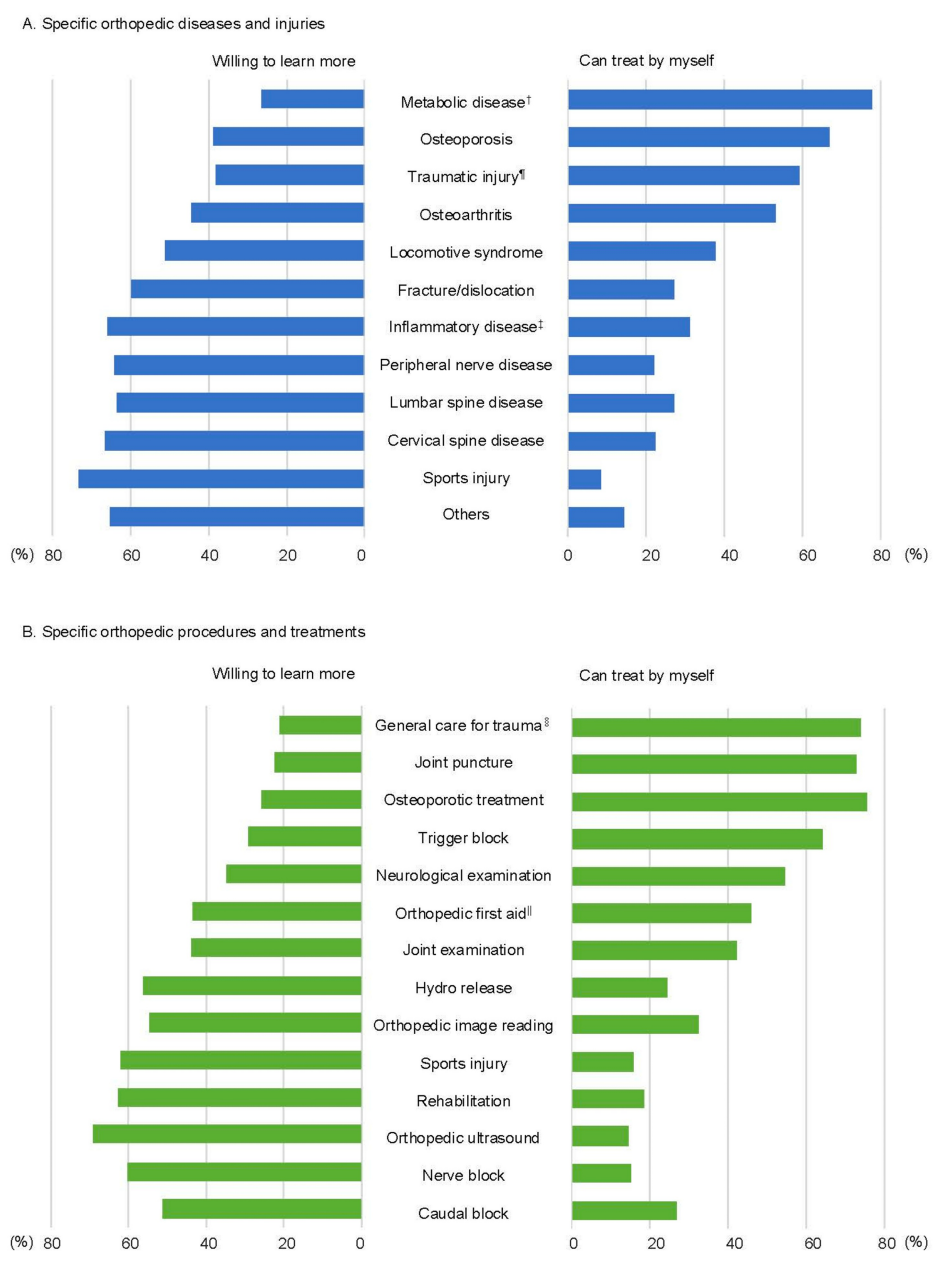
Answers for specific orthopedic practice (diseases/injuries, and procedures/treatments) in primary care settings (multiple selections). A. Specific orthopedic diseases and injuries. B. Specific orthopedic procedures and treatments. †Induced by metabolic disorders such as hyperuricemia and crystal arthritis. ¶General trauma such as incised wounds, lacerations, abrasions, and contusions. ‡Induced by general inflammatory disorders such as rheumatoid arthritis and spondyloarthritis. §Suture, debridement, drainage. ||Reduction and external fixation for fracture or dislocation.

Responses to questions about specific orthopedic practices (diseases/injuries and procedures/treatments) are shown in Figure 3. Regarding specific orthopedic diseases and injuries (Figure 3A), many PCPs reported that they could treat common conditions in internal medicine (metabolic syndrome and osteoporosis) by themselves while being unable to treat cases that potentially required expertise in orthopedics (sports injury, cervical, and lumbar spine diseases), and expressed a willingness to learn more about these diseases. Similar responses were found for orthopedic procedures and treatments (Figure 3B), indicating that PCPs performed procedures for general injuries but were not confident in treating orthopedic-specific injuries such as fractures, dislocation, and sports injuries.

## Discussion

In rural and remote settings, PCPs such as GPs and those with expertise in general internal medicine are often required to engage in comprehensive medical care, including treatments and procedures for orthopedic and musculoskeletal conditions. Additionally, they are required to respond to orthopedic-specific trauma, such as fractures, as part of first aid. This study revealed that most PCPs, especially in rural and remote areas with careers <10 years in length, frequently required orthopedic knowledge and skills in their daily practice. These PCPs expressed a strong willingness to learn orthopedic knowledge and skills and participate in orthopedic training sessions. Moreover, they were more likely to perceive a burden because of a lack of knowledge and procedural skills. When examining workplace differences, PCPs in rural and remote areas were significantly more likely to perceive burden due to a lack of knowledge, procedural skills, and longer time requirements for orthopedic practice. While many PCPs could perform common procedures such as general care for trauma, joint puncture, and trigger block, less than half reported confidence in managing orthopedic-specific injuries like fracture and dislocation. As hypothesized, PCPs may not be adequately prepared to address orthopedic and musculoskeletal problems due to limited educational opportunities. Also, the workplace differences are thought to be the limited access to orthopedic specialists in rural and remote areas, necessitating that they have to manage orthopedic-specific cases by themselves. Although this study did not assess the frequency of educational opportunities nor their recognition of satisfaction with current educational sessions, we could show the PCPs’ motivation or passion for the educational opportunities. Interestingly, differences in motivation to learn were observed depending on their years of experience and workplace even though lifelong learning was an obligation of physicians. PCPs in other areas with careers <10 years in length demonstrated significantly lower interest in orthopedic practice than other groups. PCPs with careers <10 years in length who participated in this survey were obliged to work in rural and remote areas, and most of them might tend to have expertise in general practice and general internal medicine after their obligation. Consequently, there might be a divergence between willingness and interest in orthopedics. 

Based on the results of this study, PCPs in rural and remote areas should be equipped to provide initial treatment for orthopedic-specific conditions and injuries, particularly fractures and dislocations requiring procedural skills. Particularly in rural and remote settings, trauma care is challenging because of limited human and medical resources, delays in presentation, underestimation of urgency, and deficiencies in local support services, resulting in substantial differences in treatment outcomes in different situations [20-22]. Although few reports have focused on trauma care in rural and remote areas in Japan, one study conducted on a small remote island reported that half of new patients had complaints of musculoskeletal problems, and 67 of 1404 (4.8%) new patients had orthopedic-specific injuries [9,10]. These reports support the current finding that PCPs in rural and remote areas were commonly required to treat patients with orthopedic and musculoskeletal complaints by themselves. Additionally, a few previous studies found that some PCPs had inadequate orthopedic knowledge and lacked confidence in trauma care [11,12]. Our results highlight an important issue for understanding and improving care for orthopedic-specific injuries in rural and remote areas: PCPs engaging in such areas should be provided with sufficient opportunities to learn knowledge and skills for managing orthopedic-specific injuries. 

In addressing the issues highlighted in this study, it is essential to consider the current education systems. Education for musculoskeletal medicine is underrepresented in medical school curricula internationally; the learning duration for musculoskeletal medicine is disproportionately short despite the large number of patients [23,24]. This situation might have contributed to the perceived insufficiency in knowledge and skills among the PCPs in our study, as education in musculoskeletal medicine and orthopedics was not covered in a specialized curriculum, even in comparison to other universities. A previous study reported that participation in a clinical elective program was the only factor that led to a significant increase in musculoskeletal knowledge among medical students [25]. However, it is doubtful that limited medical school curricula and immediate post-graduate training for musculoskeletal and orthopedic problems would be beneficial many years later. Therefore, education programs for PCPs like those provided in North America would be beneficial [13-15]. These programs are typically training courses conducted over several days, which consist of interactive small-group teaching, such as case discussions, exam demonstrations, and assessment of participants’ physical examinations, and can provide skills for managing musculoskeletal problems and improving participants’ confidence. However, frequent participation in such practical training sessions poses challenges for PCPs in rural and remote areas, particularly in the context of the current situation in Japan. Thus, programs with fewer time and geographical constraints should be developed and prioritized. 

There has been an increase in the use of information and communications technology to facilitate videoconferencing and telephone consultations to meet physical distancing requirements in recent years. Moreover, the coronavirus disease 2019 pandemic rapidly transformed healthcare systems worldwide with significant variations and innovations. There is increasing support for the use of information and communications technology platforms to improve the accessibility of health services, especially in high-income countries [26,27]. Implementation of rural remote telemedicine and virtual/electronic consults is reported to be beneficial for physicians when requiring emergent specialist care and may enable high-level trauma care [22,28-30]. However, securing social resources to provide these systems at night and on holidays is an important challenge, particularly in rural areas. Moreover, these new methods cannot be used to perform procedures directly, and the issue of responsibility when problems occur with remotely controlled procedures remains. Although many problems remain, such new technology can be a sufficient support for medical care and education in rural and remote areas, and it is hoped that such a system will be established in the future.

To summarize, most PCPs in rural and remote areas with careers <10 years in length required orthopedic knowledge and skills in their daily practice and were willing to learn orthopedic knowledge and skills and participate in orthopedic training sessions. PCPs in rural and remote areas with careers <10 years in length were more likely to perceive burden because of a lack of knowledge and procedural skills. Many PCPs in this study were not confident in treating orthopedic-specific injuries such as fractures and dislocations. To improve these issues, a training program to learn knowledge and skills for managing orthopedic-specific injuries might be provided for PCPs. And these programs should be provided to younger PCPs in rural and remote areas for the sake of cost-effectiveness. Moreover, it is necessary to establish a new telemedicine system that can provide musculoskeletal and orthopedic knowledge and skills to support medical care and education in rural and remote areas.

The current study involved several limitations. First, the survey participants were restricted to graduates of a single university in Japan and the response rate of the questionnaire was relatively low. As mentioned, JMU is the only medical school in Japan whose mission is to produce rural physicians, and graduates are obliged to work mainly in rural regions for nine years of their careers. There may be a problem with investigating them as representatives of Japanese PCPs. However, previous studies have reported that JMU graduates contributed significantly to rural health care in Japan [17,18]. In 2004, despite being one of 80 medical schools in Japan, 578 of 7,212 (8.0%) physicians who engaged in rural practice were JMU graduates. Also, JMU graduates were 5.9 times more likely to work in rural areas than were non-JMU graduates. Moreover, the data in this survey were collected from PCPs with different careers in 46 prefectures excluding Osaka, which had no rural or remote areas. Therefore, we think the results of this survey reflect the current state of rural and remote areas in Japan to some extent and are worth reporting. Second, our survey did not obtain information regarding the sex of the participants. Sex differences may have affected our results. Third, there were some problems with the questionnaire in this study. The questionnaire was developed by the authors because we were unable to identify similar previous studies examining this issue. As a result, it had not been evaluated for validity. Also, our survey was based on participant descriptions (self-administration), which may introduce information bias. Therefore, when interpreting and applying the results of this study, it may be necessary to take this into account.

## Conclusions

This study highlighted that PCPs in rural and remote areas, particularly those with less than 10 years of experience, often required orthopedic knowledge and skills in their daily practice and demonstrated a strong willingness to learn them. While it is desirable for these PCPs in rural and remote areas to be equipped to provide initial treatment for orthopedic-specific conditions and injuries, many of them lack confidence in managing cases such as fractures and dislocations, perceiving a burden due to insufficient knowledge and procedural skills. To address these issues, implementing targeted training programs and telemedicine systems that offer accessible and practical orthopedic and musculoskeletal education could be transformative in supporting PCPs and improving orthopedic care.
